# A physical analysis of the Y chromosome shows no additional deletions, other than Gr/Gr, associated with testicular germ cell tumour

**DOI:** 10.1038/sj.bjc.6603557

**Published:** 2007-01-09

**Authors:** R Linger, D Dudakia, R Huddart, D Easton, D T Bishop, M R Stratton, E A Rapley

**Affiliations:** 1 Testicular Cancer Genetics Team, Section of Cancer Genetics, Institute of Cancer Research, Brookes Lawley Building, Sutton, Surrey SM2 5NG, UK; 2 Academic Radiotherapy Unit, Institute of Cancer Research, Sutton, Surrey SM2 5PT, UK; 3 Genetic Epidemiology Unit, Cancer Research UK, Strangeways Research Laboratory, Cambridge CB1 8RN, UK; 4 Genetic Epidemiology Division, Cancer Research UK Clinical Centre, St James's University Hospital, Leeds LS9 7TF, UK

**Keywords:** Y chromosome, testicular cancer, gr/gr, deletion, germ cell tumour

## Abstract

Testicular germ cell tumour (TGCT) is the most common malignancy in men aged 15–45 years. A small deletion on the Y chromosome known as ‘gr/gr’ was shown to be associated with a two-fold increased risk of TGCT, increasing to three-fold in cases with a family history of TGCT. Additional deletions of the Y chromosome, known as AZFa, AZFb and AZFc, are described in patients with infertility; however, complete deletions of these regions have not been identified in TGCT patients. We screened the Y chromosome in a series of TGCT cases to evaluate if additional deletions of Y were implicated in TGCT susceptibility. Single copy Y chromosome STS markers with an average inter-marker spacing of 128 kb were examined in constitutional DNA of 271 index TGCT patients. Three markers showed evidence of deletions, sY1291, indicative of ‘gr/gr’ (eight out of 271; 2.9%), Y-DAZ3 contained within ‘gr/gr’ (21 out of 271; 7.7%) and a single deletion of the marker G66152 was identified in one TGCT case. No other markers demonstrated deletions. While several regions of the Y chromosome are known to be deleted and associated with infertility, our study provides no evidence to suggest regions of Y deletion, other than ‘gr/gr’, are associated with susceptibility to TGCT in UK patients.

Testicular germ cell tumour (TGCT) is the most common cancer in men aged 15–45 years. The worldwide incidence of the disease is 7.5 per 100 000, but the rates vary considerably between countries and racial and ethnic groups (Ferlay *et al*, 2004). The highest incidence of the disease occurs in men of European descent and the lowest in African and Asian groups (Ferlay *et al*, 2004). Risk factors for TGCT include a family history of disease (Forman *et al*, 1992; Heimdal *et al*, 1996; Westergaard *et al*, 1996; Sonneveld *et al*, 1999; Hemminki and Li, 2004), a previously diagnosed germ cell tumour (Osterlind *et al*, 1991; Wanderas *et al*, 1997), a history of undescended testis (UDT) (Brown *et al*, 1987; Swerdlow *et al*, 1997), infertility (Petersen *et al*, 1998; Moller and Skakkebaek, 1999; Jacobsen *et al*, 2001; Richiardi *et al*, 2004), atrophy (Harland *et al*, 1998) and gonadal dysgenesis (Verp and Simpson, 1987).

Family history is one of the strongest of the underlying risk factors. Multiple studies have documented that brothers and fathers of patients with TGCT have a 8–12-fold and 4–6-fold risk, respectively (Forman *et al*, 1992; Heimdal *et al*, 1996; Westergaard *et al*, 1996; Sonneveld *et al*, 1999; Hemminki and Li, 2004). These relative risks are higher than for most other cancer types, which rarely exceed 4 (Hemminki *et al*, 2001) and suggest that there is a substantial contribution to the disease risk from underlying susceptibility genes.

Despite the high familial risk, the underlying genetic susceptibility to the TGCT remains unclear. A recent genome-wide linkage search on 237 TGCT pedigrees, the largest series of TGCT families examined to date, identified several ‘regions of interest’ on chromosomes 2p33, 3p12, 3q26, 12q13–q21, 18q21–q23 and Xq27. Each region demonstrated a HLOD score of greater than 1 but not exceeding 2. The study showed that susceptibility to TGCT could not be accounted for by a single major gene or even two major genes (like for example *BRCA1* or *BRCA2* in breast cancer) and was more likely to be due to several genes with modest or small effects on risk (Crockford *et al*, 2006).

Recently, we and others examined the ‘gr/gr’ deletion on the Y chromosome in a series of 4441 TGCT cases and control males and showed that this 1.6 Mb deletion was associated with an two-fold increased risk of TGCT, increasing to three-fold in patients with a family history of TGCT, suggesting that this deletion is a rare, low penetrance allele conferring susceptibility to TGCT (Nathanson *et al*, 2005).

The ‘male-specific region’ (MSY) of the Y chromosome contains an ampliconic region made up of eight massive palindromes, which show greater that 99.9% homology. Large deletions of the ampliconic region of the Y known as AZFa, -b and -c (MIM 415000) have been described in patients with infertility. These deletions generally remove all copies of a specific gene family resulting in complete infertility (Vogt *et al*, 1996; Kuroda-Kawaguchi *et al*, 2001). The smaller ‘gr/gr’ deletion, which lies within the AZFc region, removes some but not all of the testis-specific Y gene copies, and transmission of this deletion from father to son has been described (Repping *et al*, 2003). Several studies have examined constitutional DNA from TGCT patients for deletions of the AZF regions on the Y chromosome. A total of 457 TGCT patients were examined and no deletions were identified in the AZF regions (Frydelund-Larsen *et al*, 2003; Lutke Holzik *et al*, 2005; Bor *et al*, 2006). However, the STS markers used for the analysis would not detect the ‘gr/gr’ deletion. Two further studies have examined the AZF regions in a series of TGCT patients in constitutional and tumour material, reporting a high frequency of deletions in normal and tumour samples. In a series of 17 Finnish men, 76.4% showed deletions of between one and eight STS markers and in 40 TGCT cases from Norway and Argentina, 25% of cases showed a deletion of at least one STS marker. Interestingly, none of these deletions showed a contiguous pattern with more than two STS markers deleted. Furthermore, the deletions or absence of an STS marker in constitutional DNA was shown to be present in the corresponding tumour material of cases. It is difficult, therefore, to conclude if these are real Y microdeletions or if the deletions are due to some form of mosaicism as the authors suggest (Bianchi *et al*, 2002, 2006; Richard *et al*, 2004).

We considered that additional as yet unidentified deletions of the Y chromosome, particularly in the ampliconic region of MSY, could also be implicated in TGCT susceptibility and therefore examined the Y chromosome in the constitutional DNA of a series of TGCT cases with a fine STS marker map to determine if additional regions of deletion could be implicated in the disease.

## METHODS

Testicular germ cell tumour patient samples were collected from UK oncology centres as part of a study into genetic susceptibility to TGCT. Patients donated samples and medical information with full informed consent and with local or national ethical review board approval. Information on clinical status, including type of TGCT, age of diagnosis, presence of UDT and laterality of disease, was confirmed by reviewing histological reports and clinical notes. DNA was extracted from whole blood using standard techniques. Samples were allocated to three analysis groups: ‘family history’, patients with TGCT and another family member affected with TGCT; ‘sporadic’, TGCT patients with no family history of disease and ‘UDT’, TGCT cases without a family history of TGCT but with a family history of UDT. Control male DNA samples were purchased from the European Collection of Cell Cultures (ECACC). All control samples and TGCT samples were from white UK males; however, cases and controls were not age matched.

Markers were selected from the NCBI database (http://www.ncbi.nlm.nih.gov/). A marker was selected if it existed in a single copy on the Y chromosome sequence and if there was specific sequence map location (*Homo sapiens* build 35.1). A total 192 Y chromosome STS markers were selected to be evenly spaced from the p to q arm. All samples were checked for the presence of the *SRY* gene (genBank accession number=G38356).

The STS markers were amplified in a single-plex reaction containing 25 ng genomic DNA using standard conditions. Polymerase chain reaction products were run on 2% agarose gels and visualised by staining with ethidium bromide. Samples were scored as positive if a PCR fragment at the correct size was seen on the gel, and negative if no PCR product was seen. For all markers other than sY1291 and Y-DAZ3, negative samples were repeated in a single-plex reaction and in a multiplex reaction with the *SRY* gene acting as a positive control. If samples remained negative, the primers for the STS marker were redesigned so that the new primer sites flanked the existing STS. The newly designed primer pairs were retested on previously negative samples in both single-plex and multiplex reactions ensuring that the deletion was not due to a polymorphism within the original primer site. Only if the sample remained negative after amplification of the modified primer pairs was the sample scored as deleted. For sY1291 and Y-DAZ3, the repetitive nature of the Y chromosome sequence in these regions prevented the design of flanking primers that only amplified a single copy of the Y sequence. Deletions of sY1291 were confirmed by a multiplex reaction as previously described (Nathanson *et al*, 2005). Deletions of Y-DAZ3 were confirmed by a single-plex reaction and two multiplex reactions, one with *SRY* acting as the positive control and one with sY1240 as a positive control.

## RESULTS

### Patient series

Two hundred and seventy-one samples were examined in this study, of which 167 cases had TGCT and a family history of disease (family history group), 96 patients had TGCT and no family history (sporadic group) and eight cases had TGCT and a family history of UDT (UDT group) (Table 1a). All patients had TGCT except for five, who were diagnosed with extragonadal germ cell tumours. Eighteen patients had bilateral disease, 12 in the family history group and six in the sporadic group. The pedigree structures, histologies and age of diagnosis for the series is shown in Tables 1a and 1b.

### Y STS marker map

The average marker spacing of the single copy Y STS markers is 128 kb. The list of the STS markers used in the analysis can be found in the Supplementary Table. Four regions had inter-marker distances of greater than 1 Mb (largest 1.55 Mb). These regions were at 4.1 Mb (DYS253) – 5.3 Mb (sY1240); 11.2 Mb (sY1200) – 12.2 Mb (G65937), 23.8 Mb (sY1291) – 25.2 Mb (Y-DAZ3) and 25.2 Mb (Y-DAZ3) – 26.7 Mb (sY1201) along the Y chromosome sequence. Single copy STS markers could not be identified within these regions from the public databases. An additional seven regions had inter-marker distances of greater than 0.5 Mb.

Seven STS markers initially gave negative results across multiple samples. Each of these STS had the primers redesigned so that the new primer pair flanked the old pair. After amplification with the new primers, all samples, except in one case as reported below, demonstrated a PCR product of the correct size indicating that the STS was not truly deleted and that the original nonamplification was likely to be due to a polymorphism in the original primer sites.

### STS marker deletion analysis

All samples were investigated for the presence of the *SRY* gene and all samples were positive for this marker. One hundred and ninety-two single copy STS Y markers were examined in all samples.

One STS, G66152, showed a single deletion in one TGCT case. The sample remained negative in multiplex analysis and also once the primers were redesigned flanking the original primer sites. The patient had a right-sided non-seminoma at age 31 years and no family history of TGCT. He also had an operation for an undescended testis on the left side at age 8 years. The patient is considered ‘fertile’ as he has one child. There was no DNA available from other family members to determine if this deletion was shared with other male relatives.

Two STS markers sY1291 (GenBank Accession Number G72340) and Y-DAZ3 (G73170) were deleted in multiple samples. sY1291 is the marker used to indicate a gr/gr deletion and eight samples (Table 2) demonstrated a deletion in this region. The frequency of gr/gr in this series was 2.9%. All samples demonstrating a gr/gr deletion have been reported previously in Nathanson *et al* (2005). Twenty-one of 271 (7.7%) TGCT cases showed deletions in Y-DAZ3 (Table 2). Examination of this region in a series of 399 control male samples from the ECACC showed 30 deletions (7.5%) (*P*=1.0, Fishers exact test). Only three TGCT cases and two controls demonstrated deletions for both sY1291 and Y-DAZ3 (Table 2). Owing to the repetitive nature of the Y chromosome sequence in this region, neither of these STS could be redesigned with flanking primers and ensure that the STS remained single copy. However, the analysis in both single-plex and multiplex reactions was repeated at least three times and remained negative.

Of the 21 TGCT cases with Y-DAZ3 deletions, 10 cases had a seminoma (46%), seven cases had a non-seminoma (33%) and four (19%) had a mixed histology tumour. Two cases, one with a family history and one without, had bilateral disease. The average age of diagnosis of the first tumour for the group was 29.25 years. Fertility status was unknown, but only five of 21 (24%) cases reported having children.

In TGCT cases with a ‘gr/gr’ deletion as characterised by a negative result at sY1291, six cases had a seminoma (75%), one a non-seminoma (12.5%) and one case had a mixed histology tumour (12.5%). The ‘gr/gr’ deletion has previously been shown to be more strongly associated with seminoma (Nathanson *et al*, 2005). Four of eight cases reported having children.

Markers spanning the AZFa, AZFb and AZFc regions of the Y chromosome, frequently deleted in patients with infertility, were examined by markers spanning and flanking these regions. The AZFa region was evaluated by 15 single copy STS markers. The AZFb and AZFc regions, which exhibit a degree of overlap, were evaluated with 46 single copy STS (Supplementary Table). No deletions were demonstrated in either the AZFa or AZFb region. The AZFc region contains the 1.6 Mb gr/gr region and the only deletions observed in this series were in the ‘gr/gr’ region, and there was no evidence of the larger AZFc deletion.

## DISCUSSION

Having previously identified an association between the Y chromosome ‘gr/gr’ deletion and TGCT, a physical analysis of the Y chromosome was conducted to determine if additional regions of the Y chromosome are implicated in TGCT susceptibility, focusing particularly on the MSY region. Our analysis showed that other than the ‘gr/gr’ deletion, no other deletions of Y are likely to be associated with TGCT risk. Although it is possible that we may have missed small regions of deletions, particularly in those few regions where the inter-marker spacing exceeds 1 Mb, none of our inter-marker distances exceeds that of deletions previously described for the Y, including the ‘gr/gr deletion.

Previous studies of Y chromosome deletions in TGCT has focused on the AZF deletion regions initially identified in patients with infertility. Many of the markers used in previous studies have also been utilised for this study. Our study differs from previous studies in that we examined these regions in more detail by using a higher density of markers. Similar to the previous studies in Danish and Norwegian TGCT patients (Frydelund-Larsen *et al*, 2003; Lutke Holzik *et al*, 2005; Bor *et al*, 2006), no large contiguous deletions were demonstrated for any sample in this series in the AZF regions. We did not see the rate of noncontiguous deletions in the AZF regions demonstrated previously for Finnish patients and Argentinean TGCT patients (Bianchi *et al*, 2002, 2006; Richard *et al*, 2004), suggesting that there is either a difference in the rate of deletion between these populations or that there is some methodological issue giving rise to nonamplification of a PCR product rather that a true deletion of the Y chromosome DNA in these studies. None of the previous studies of the AZF regions and TGCT used STSs that would have detected the ‘gr/gr’ deletion.

We took considerable care to confirm all negative results. Samples that initially showed a negative PCR reaction were repeated in both a single-plex and a multiplex reaction, with SRY as the positive control. If the sample remained negative in these reactions, we redesigned the primer sites to flank the original primer, thus eliminating the possibility of a polymorphism in the primer site causing the nonamplification of the fragment. Only for sY1291 and Y-DAZ3 was this procedure not possible due to the degree of sequence identity in these regions with other Y regions. However, these results were repeated on numerous occasions (at least three times) in multiplex analyses and remained negative. We are therefore confident that where a fragment failed to amplify that it represented a true deletion on the Y chromosome DNA at that position. The patient series in this study is large, well characterised and includes patients both with and without a family history of disease. All patients were white Caucasian males from the UK and the series is representative of the ethnic group with one of the highest incidences of TGCT. The patient series may not be sufficiently large to detect very rare deletion variants, however these rare variants would have little impact on overall susceptibility to TGCT.

**Figure 1:**
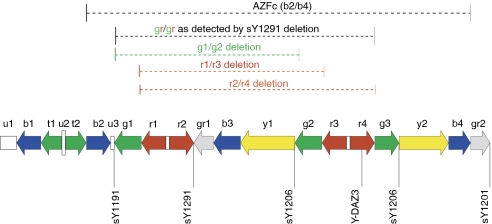
Schematic of the AZFc deletion region showing gr/gr deletion and mechanisms whereby this deletion can arise, adapted from Nathanson *et al*, 2005.

Eight of the patient series showed deletions of the ‘gr/gr’ region as characterised by the absence of the marker sY1291. All patients in the present study with ‘gr/gr’ have been reported previously as part of a much larger series (Nathanson *et al*, 2005). The ‘gr/gr’ deletion as detected by the absence of the marker sY1291 can be generated by a number of different Y rearrangements (Figure 1). We identified three TGCT patients and two control patients with deletions of both sY1291 and Y-DAZ3, which might suggest that these variants arose via the r1/r4 mechanism (Figure 1). All other ‘gr/gr’ deletions did not have an accompanying deletion of Y-DAZ3, which could implicate that these have arisen via the g1/g2 or r1/r3 mechanism (Figure 1). This observation suggests that further characterisation of the ‘gr/gr’ deletion region is needed in TGCT and this may provide information on the genes in this region that are critical for TGCT susceptibility.

We observed that the frequency of Y-DAZ3 deletions alone was similar among cases and controls. Both the patient data set and the control series were all white Caucasian males from the UK. The control series, while not collected in a formal case–control fashion, would be expected to match the ethnic background of the case series. This suggests that the deletion at Y-DAZ3 is a neutral polymorphism in this population and not implicated in susceptibility to TGCT. Analysis of SNV variants and the Y-DAZ3 marker have suggested that deletions of DAZ3/4 are associated with Y haplogroup N, which is common among Northern Europeans (Fernandes *et al*, 2004). It is unknown if the Y-DAZ3 deletion exists on the same Y haplotype among this series, as Y chromosome haplotyping was not performed as part of this study.

A single sample demonstrated a deletion at the STS marker G66152, both the flanking STS markers, sY1252 (196 kb away) and sY1253 (51 kb away), were positive. As there are no genes identified in this region, it is unlikely that the small deletion is involved in pathogenesis of TGCT.

We have previously shown that the ‘gr/gr’ deletion is associated with a two-fold risk of TGCT increasing to three-fold in TGCT cases with a family history of disease. We evaluated the Y chromosome with a dense marker map and no significant additional deletion regions of the Y chromosome were detected. Excluding the possibility of small deletions existing between the STS markers used or very rare deletions of Y that may not have been detected in this series, the study showed that other than the previously characterised ‘gr/gr’ deletion, Y chromosome deletions do not make a significant contribution to TGCT susceptibility.

## Figures and Tables

**Table 1a:** Characteristics of TGCT samples used in study – pedigree structure

**Group**	**Family structure**	**Number of index cases per family type**	**Total number of TGCT cases**
Family history			167
	Sib pair	75	
	Sib trio	2	
	Father/son pair	28	
	Cousin pair	25	
	Large >3 affected cases	11	
	Uncle nephew pair	18	
	Grandfather/grandson	4	
	Twins (MZ)	4	
			
Sporadic			96
			
UDT			8
			
Total			271

GCT=testicular germ cell tumour; UDT=undescended testis.

**Table 1b:** Characteristics of TGCT samples used in study – histology and age of diagnosis (of first tumour)

	**Seminoma**		**Non-seminoma**		**Mixed histology**		**Unknown**		**All histology (and unknown histology)**	
**Group**	**Number of cases**	**Age of diagnosis**	**Number of cases**	**Age of diagnosis**	**Number of cases**	**Age of diagnosis**	**Number of cases**	**Age of diagnosis**	**Number of cases**	**Age of diagnosis**
Family history	81 (0.52)	37.22	65 (0.41)	28.14	11 (0.07)	34.54	10	29.22	167	33.08
Sporadic	38 (0.40)	35.5	42 (0.45)	30.23	14 (0.15)	31.43	2	47.5	96	32.85
UDT	6 (0.86)	28.16	0 (0.00)	—	1 (0.14)	45	1	42	8	32
										
Total	125	36.26	107	28.97	26	33.26	13	33.97	271	32.97

GCT=testicular germ cell tumour; UDT=undescended testis.

**Table 2:** Deletions in sY1291 and Y-DAZ3

**Group**	**Family history**	**OR**	**Sporadic**	**OR**	**UDT**	**OR**	**All TGCT cases**	**OR**	**Controls**
sY1291 (gr/gr)	4/167 (0.024)	1.61 (0.33, 6.87)	4/96 (0.04)	2.85 (0.58, 12.26)	0/8	0.0 (0.0, 34.6)	8/271 (0.029)	1.99 (0.60, 7.04)	6/399 (0.015)
Y-DAZ3	12/167 (0.072)	0.95 (0.43, 1.98)	9/96 (0.094)	1.27 (0.51, 2.87)	0/8	0.0 (0.0, 6.08)	21/271 (0.077)	1.03 (0.55, 1.91)	30/399 (0.075)
Both sY1291 and Y-DAZ3 deletion	2/167 (0.012)	2.41 (0.17, 33.4)	1/96 (0.010)	2.09 (0.04, 40.5)	0/8	0.0 (0.0, 106.5)	3/271 (0.011)	2.22 (0.25, 26.73)	2/399 (0.005)
Y-DAZ3 deletion only	10/167 (0.060)	0.84 (0.36, 1.84)	8/96 (0.081)	1.20 (0.46, 2.83)	0/8	0.0 (0.0, 6.56)	18/271 (0.079)	0.94 (0.48, 1.81)	28/399 (0.103)

The table reports the number of samples showing the deletion compared to the number tested (and the proportion) plus the odds ratio (OR) for the comparison to the controls (and 95% confidence interval) for the family history positive group and the family history negative group.
