# Supplementary material for: A physical analysis of the Y chromosome shows no additional deletions, other than Gr/Gr, associated with testicular germ cell tumour
Source: Br J Cancer. 2007 Jan 9;96(2):357–61. doi: 10.1038/sj.bjc.6603557 (PMC2360005; doi:10.1038/sj.bjc.6603557)
Supplement: Supplementary Table [file 6603557x1.doc]

Supplementary Table: Y STS markers used to evaluate deletions on the Y chromosome.

| 4 |  |  |  |  |  | Family History | | Sporadic | | UDT | | Total | |
| --- | --- | --- | --- | --- | --- | --- | --- | --- | --- | --- | --- | --- | --- |
|  |  | Marker Name | UniSTS | Position on Y chromosome (Kb) | inter-marker distance (kb) | number of deletions | Frequency | number of deletions | Frequency | number of deletions | Frequency | Number of deletions | Frequency |
|  | ϕΨξ | SRY | 42547 | 2698 |  | 0 | 0.00 | 0 | 0.00 | 0 | 0.00 | 0 | 0 |
|  |  | RH38681 | 89213 | 2698 | 0 | 0 | 0.00 | 0 | 0 | 0 | 0.00 | 0 | 0 |
|  |  | DYS376 | 149030 | 2706 | 8 | 0 | 0.00 | 0 | 0 | 0 | 0.00 | 0 | 0 |
|  |  | SHGC-1318 | 21892 | 2752 | 46 | 0 | 0.00 | 0 | 0 | 0 | 0.00 | 0 | 0 |
|  |  | DYS250 | 25585 | 2762 | 10 | 0 | 0.00 | 0 | 0 | 0 | 0.00 | 0 | 0 |
|  |  | G65821 | 230196 | 2808 | 46 | 0 | 0.00 | 0 | 0 | 0 | 0.00 | 0 | 0 |
|  |  | RH38667 | 90503 | 2866 | 58 | 0 | 0.00 | 0 | 0 | 0 | 0.00 | 0 | 0 |
|  |  | DYS252 | 1011 | 2957 | 91 | 0 | 0.00 | 0 | 0 | 0 | 0.00 | 0 | 0 |
|  |  | DYS395 | 58494 | 3174 | 217 | 0 | 0.00 | 0 | 0 | 0 | 0.00 | 0 | 0 |
|  |  | G66071 | 230062 | 3893 | 719 | 0 | 0.00 | 0 | 0 | 0 | 0.00 | 0 | 0 |
|  |  | DYS253 | 10646 | 4121 | 228 | 0 | 0.00 | 0 | 0 | 0 | 0.00 | 0 | 0 |
|  |  | sY1240 | 256778 | 5309 | 1188 | 0 | 0.00 | 0 | 0 | 0 | 0.00 | 0 | 0 |
|  |  | G65945 | 230541 | 5468 | 159 | 0 | 0.00 | 0 | 0 | 0 | 0.00 | 0 | 0 |
|  |  | sY1241 | 256779 | 6146 | 678 | 0 | 0.00 | 0 | 0 | 0 | 0.00 | 0 | 0 |
|  |  | G65838 | 230504 | 6660 | 514 | 0 | 0.00 | 0 | 0 | 0 | 0.00 | 0 | 0 |
|  |  | G66289 | 230130 | 6699 | 39 | 0 | 0.00 | 0 | 0 | 0 | 0.00 | 0 | 0 |
|  |  | G38362 | 32711 | 6780 | 81 | 0 | 0.00 | 0 | 0 | 0 | 0.00 | 0 | 0 |
|  |  | DYS265 | 30765 | 6812 | 32 | 0 | 0.00 | 0 | 0 | 0 | 0.00 | 0 | 0 |
|  |  | DYS266 | 40588 | 6849 | 37 | 0 | 0.00 | 0 | 0 | 0 | 0.00 | 0 | 0 |
|  |  | SHGC-110324 | 167773 | 6903 | 54 | 0 | 0.00 | 0 | 0 | 0 | 0.00 | 0 | 0 |
|  |  | G66293 | 230134 | 6953 | 50 | 0 | 0.00 | 0 | 0 | 0 | 0.00 | 0 | 0 |
|  |  | sY1321 | 259324 | 7001 | 48 | 0 | 0.00 | 0 | 0 | 0 | 0.00 | 0 | 0 |
|  |  | G66010 | 230606 | 7217 | 216 | 0 | 0.00 | 0 | 0 | 0 | 0.00 | 0 | 0 |
|  |  | DYS264 | 51242 | 7281 | 64 | 0 | 0.00 | 0 | 0 | 0 | 0.00 | 0 | 0 |
|  |  | G66301 | 230142 | 7295 | 14 | 0 | 0.00 | 0 | 0 | 0 | 0.00 | 0 | 0 |
|  |  | DYS263 | 31231 | 7335 | 40 | 0 | 0.00 | 0 | 0 | 0 | 0.00 | 0 | 0 |
|  |  | DYS262 | 9249 | 7381 | 46 | 0 | 0.00 | 0 | 0 | 0 | 0.00 | 0 | 0 |
|  |  | G66283 | 230124 | 7430 | 49 | 0 | 0.00 | 0 | 0 | 0 | 0.00 | 0 | 0 |
|  |  | G66286 | 230127 | 7469 | 39 | 0 | 0.00 | 0 | 0 | 0 | 0.00 | 0 | 0 |
|  |  | sY1219 | 256773 | 7516 | 47 | 0 | 0.00 | 0 | 0 | 0 | 0.00 | 0 | 0 |
|  |  | DYS260 | 9849 | 7627 | 111 | 0 | 0.00 | 0 | 0 | 0 | 0.00 | 0 | 0 |
|  |  | DYS288 | 28575 | 7701 | 74 | 0 | 0.00 | 0 | 0 | 0 | 0.00 | 0 | 0 |
|  |  | G65974 | 230570 | 7792 | 91 | 0 | 0.00 | 0 | 0 | 0 | 0.00 | 0 | 0 |
|  |  | DYS54 | 149019 | 7841 | 49 | 0 | 0.00 | 0 | 0 | 0 | 0.00 | 0 | 0 |
|  |  | G66004 | 230600 | 7960 | 119 | 0 | 0.00 | 0 | 0 | 0 | 0.00 | 0 | 0 |
|  |  | SHGC-107423 | 170353 | 8044 | 84 | 0 | 0.00 | 0 | 0 | 0 | 0.00 | 0 | 0 |
|  |  | G66121 | 229960 | 8151 | 107 | 0 | 0.00 | 0 | 0 | 0 | 0.00 | 0 | 0 |
|  |  | G66127 | 229966 | 8170 | 19 | 0 | 0.00 | 0 | 0 | 0 | 0.00 | 0 | 0 |
|  |  | G65901 | 230646 | 8364 | 194 | 0 | 0.00 | 0 | 0 | 0 | 0.00 | 0 | 0 |
|  |  | G66264 | 230105 | 8500 | 136 | 0 | 0.00 | 0 | 0 | 0 | 0.00 | 0 | 0 |
|  |  | G65900 | 230645 | 8527 | 27 | 0 | 0.00 | 0 | 0 | 0 | 0.00 | 0 | 0 |
|  |  | G66104 | 229943 | 8618 | 91 | 0 | 0.00 | 0 | 0 | 0 | 0.00 | 0 | 0 |
|  |  | G66016 | 230612 | 8945 | 327 | 0 | 0.00 | 0 | 0 | 0 | 0.00 | 0 | 0 |
|  |  | sY1209 | 256769 | 8953 | 8 | 0 | 0.00 | 0 | 0 | 0 | 0.00 | 0 | 0 |
|  |  | sY1215 | 256772 | 9017 | 64 | 0 | 0.00 | 0 | 0 | 0 | 0.00 | 0 | 0 |
|  |  | G66107 | 229946 | 9142 | 125 | 0 | 0.00 | 0 | 0 | 0 | 0.00 | 0 | 0 |
|  |  | GDB:187630 | 2012 | 9969 | 827 | 0 | 0.00 | 0 | 0 | 0 | 0.00 | 0 | 0 |
|  |  | sY1243 | 256781 | 10059 | 90 | 0 | 0.00 | 0 | 0 | 0 | 0.00 | 0 | 0 |
|  |  | DYS19 | 148192 | 10115 | 56 | 0 | 0.00 | 0 | 0 | 0 | 0.00 | 0 | 0 |
|  |  | sY1244 | 256782 | 10350 | 235 | 0 | 0.00 | 0 | 0 | 0 | 0.00 | 0 | 0 |
|  |  | 6987 | 254155 | 10446 | 96 | 0 | 0.00 | 0 | 0 | 0 | 0.00 | 0 | 0 |
|  |  | G66307 | 230148 | 10520 | 74 | 0 | 0.00 | 0 | 0 | 0 | 0.00 | 0 | 0 |
|  |  | DYS269 | 77582 | 10573 | 53 | 0 | 0.00 | 0 | 0 | 0 | 0.00 | 0 | 0 |
|  |  | PMC22445P1 | 272108 | 10601 | 28 | 0 | 0.00 | 0 | 0 | 0 | 0.00 | 0 | 0 |
|  |  | sY1200 | 256768 | 11230 | 629 | 0 | 0.00 | 0 | 0 | 0 | 0.00 | 0 | 0 |
|  |  | G65937 | 229926 | 12237 | 1007 | 0 | 0.00 | 0 | 0 | 0 | 0.00 | 0 | 0 |
|  |  | DYS270 | 78323 | 12300 | 63 | 0 | 0.00 | 0 | 0 | 0 | 0.00 | 0 | 0 |
|  |  | sY1251 | 256787 | 12309 | 9 | 0 | 0.00 | 0 | 0 | 0 | 0.00 | 0 | 0 |
|  |  | DYS381 | 149036 | 12328 | 19 | 0 | 0.00 | 0 | 0 | 0 | 0.00 | 0 | 0 |
|  |  | SHGC-5864 | 5509 | 12352 | 24 | 0 | 0.00 | 0 | 0 | 0 | 0.00 | 0 | 0 |
|  | ϕξ | DYS271 | 14871 | 12535 | 183 | 0 | 0.00 | 0 | 0 | 0 | 0.00 | 0 | 0 |
|  |  | DYS391 | 36284 | 12541 | 6 | 0 | 0.00 | 0 | 0 | 0 | 0.00 | 0 | 0 |
|  |  | G66184 | 230174 | 12641 | 100 | 0 | 0.00 | 0 | 0 | 0 | 0.00 | 0 | 0 |
|  |  | G66182 | 230172 | 12674 | 33 | 0 | 0.00 | 0 | 0 | 0 | 0.00 | 0 | 0 |
|  |  | DYS272 | 18668 | 12767 | 93 | 0 | 0.00 | 0 | 0 | 0 | 0.00 | 0 | 0 |
|  |  | G42673 | 105153 | 12818 | 51 | 0 | 0.00 | 0 | 0 | 0 | 0.00 | 0 | 0 |
|  |  | DYS11 | 40309 | 12879 | 61 | 0 | 0.00 | 0 | 0 | 0 | 0.00 | 0 | 0 |
|  |  | G64723^φ^ | 181851 | 12881 | 2 | 0 | 0.00 | 0 | 0 | 0 | 0.00 | 0 | 0 |
| AZFa |  | AZFa-Prox2^φ^ | 240924 | 12881 | 0 | 0 | 0.00 | 0 | 0 | 0 | 0.00 | 0 | 0 |
|  |  | G66179 | 230169 | 12923 | 42 | 0 | 0.00 | 0 | 0 | 0 | 0.00 | 0 | 0 |
|  |  | SHGC-3904 | 47717 | 12940 | 17 | 0 | 0.00 | 0 | 0 | 0 | 0.00 | 0 | 0 |
|  |  | G49201 | 163235 | 13013 | 73 | 0 | 0.00 | 0 | 0 | 0 | 0.00 | 0 | 0 |
|  | ϕΨξ | DYS148 | 57706 | 13046 | 33 | 0 | 0.00 | 0 | 0 | 0 | 0.00 | 0 | 0 |
|  | ξ | DYS274 | 73240 | 13077 | 31 | 0 | 0.00 | 0 | 0 | 0 | 0.00 | 0 | 0 |
|  |  | RH92730 | 85641 | 13239 | 162 | 0 | 0.00 | 0 | 0 | 0 | 0.00 | 0 | 0 |
|  |  | M235 | 260448 | 13271 | 32 | 0 | 0.00 | 0 | 0 | 0 | 0.00 | 0 | 0 |
|  |  | G64992 | 164942 | 13307 | 36 | 0 | 0.00 | 0 | 0 | 0 | 0.00 | 0 | 0 |
|  |  | G66199 | 230187 | 13360 | 53 | 0 | 0.00 | 0 | 0 | 0 | 0.00 | 0 | 0 |
|  |  | G34990 | 28711 | 13470 | 110 | 0 | 0.00 | 0 | 0 | 0 | 0.00 | 0 | 0 |
|  |  | G66202 | 230190 | 13533 | 63 | 0 | 0.00 | 0 | 0 | 0 | 0.00 | 0 | 0 |
|  |  | G49212 | 163236 | 13632 | 99 | 0 | 0.00 | 0 | 0 | 0 | 0.00 | 0 | 0 |
|  |  | G64724 | 181852 | 13661 | 29 | 0 | 0.00 | 0 | 0 | 0 | 0.00 | 0 | 0 |
|  |  | AZFa-dist4 | 240919 | 13678 | 17 | 0 | 0.00 | 0 | 0 | 0 | 0.00 | 0 | 0 |
|  |  | G65231 | 166419 | 13874 | 196 | 0 | 0.00 | 0 | 0 | 0 | 0.00 | 0 | 0 |
|  |  | M231 | 260445 | 13908 | 34 | 0 | 0.00 | 0 | 0 | 0 | 0.00 | 0 | 0 |
|  |  | G65227 | 166414 | 13947 | 39 | 0 | 0.00 | 0 | 0 | 0 | 0.00 | 0 | 0 |
|  |  | DYS276 | 80503 | 14042 | 95 | 0 | 0.00 | 0 | 0 | 0 | 0.00 | 0 | 0 |
|  |  | G66221 | 230209 | 14130 | 88 | 0 | 0.00 | 0 | 0 | 0 | 0.00 | 0 | 0 |
|  |  | G66224 | 230212 | 14189 | 59 | 0 | 0.00 | 0 | 0 | 0 | 0.00 | 0 | 0 |
|  |  | DYS277 | 39528 | 14225 | 36 | 0 | 0.00 | 0 | 0 | 0 | 0.00 | 0 | 0 |
|  |  | RH45846 | 90518 | 14256 | 31 | 0 | 0.00 | 0 | 0 | 0 | 0.00 | 0 | 0 |
|  |  | DYS278 | 27109 | 14306 | 50 | 0 | 0.00 | 0 | 0 | 0 | 0.00 | 0 | 0 |
|  |  | 6371C8 | 254153 | 14347 | 41 | 0 | 0.00 | 0 | 0 | 0 | 0.00 | 0 | 0 |
|  |  | SHGC-78698 | 170073 | 14418 | 71 | 0 | 0.00 | 0 | 0 | 0 | 0.00 | 0 | 0 |
|  | ϕξ | sY182 | 64826 | 14419 | 1 | 0 | 0.00 | 0 | 0 | 0 | 0.00 | 0 | 0 |
|  |  | SHGC-5510^φ^ | 83801 | 14459 | 40 | 0 | 0.00 | 0 | 0 | 0 | 0.00 | 0 | 0 |
|  |  | G66101 | 229940 | 14531 | 72 | 0 | 0.00 | 0 | 0 | 0 | 0.00 | 0 | 0 |
|  |  | sY1306 | 256810 | 14573 | 42 | 0 | 0.00 | 0 | 0 | 0 | 0.00 | 0 | 0 |
|  |  | G66100 | 229939 | 14610 | 37 | 0 | 0.00 | 0 | 0 | 0 | 0.00 | 0 | 0 |
|  |  | G66238 | 230226 | 14639 | 29 | 0 | 0.00 | 0 | 0 | 0 | 0.00 | 0 | 0 |
|  |  | G66237 | 230225 | 14695 | 56 | 0 | 0.00 | 0 | 0 | 0 | 0.00 | 0 | 0 |
|  |  | G66232 | 230220 | 14752 | 57 | 0 | 0.00 | 0 | 0 | 0 | 0.00 | 0 | 0 |
|  |  | G65906 | 230651 | 14882 | 130 | 0 | 0.00 | 0 | 0 | 0 | 0.00 | 0 | 0 |
|  |  | SHGC-106575 | 169934 | 14951 | 69 | 0 | 0.00 | 0 | 0 | 0 | 0.00 | 0 | 0 |
|  |  | RH122991 | 135383 | 15590 | 639 | 0 | 0.00 | 0 | 0 | 0 | 0.00 | 0 | 0 |
|  |  | DYS390 | 63004 | 15713 | 123 | 0 | 0.00 | 0 | 0 | 0 | 0.00 | 0 | 0 |
|  |  | G65990 | 230586 | 15770 | 57 | 0 | 0.00 | 0 | 0 | 0 | 0.00 | 0 | 0 |
|  |  | SHGC-132887 | 170940 | 16008 | 238 | 0 | 0.00 | 0 | 0 | 0 | 0.00 | 0 | 0 |
|  |  | SHGC-85048 | 170098 | 16040 | 32 | 0 | 0.00 | 0 | 0 | 0 | 0.00 | 0 | 0 |
|  |  | G65853 | 230519 | 16102 | 62 | 0 | 0.00 | 0 | 0 | 0 | 0.00 | 0 | 0 |
|  |  | DYF135S1 | 149024 | 16116 | 14 | 0 | 0.00 | 0 | 0 | 0 | 0.00 | 0 | 0 |
|  |  | DYS243 | 49711 | 16185 | 69 | 0 | 0.00 | 0 | 0 | 0 | 0.00 | 0 | 0 |
|  |  | G65956 | 230552 | 16390 | 205 | 0 | 0.00 | 0 | 0 | 0 | 0.00 | 0 | 0 |
|  |  | G65955 | 230551 | 16398 | 8 | 0 | 0.00 | 0 | 0 | 0 | 0.00 | 0 | 0 |
|  |  | sY1305 | 256809 | 16455 | 57 | 0 | 0.00 | 0 | 0 | 0 | 0.00 | 0 | 0 |
|  |  | G66015 | 230611 | 16506 | 51 | 0 | 0.00 | 0 | 0 | 0 | 0.00 | 0 | 0 |
|  |  | G48216 | 95313 | 16568 | 62 | 0 | 0.00 | 0 | 0 | 0 | 0.00 | 0 | 0 |
|  | ξ | DYS196 | 60239 | 16633 | 65 | 0 | 0.00 | 0 | 0 | 0 | 0.00 | 0 | 0 |
|  |  | sY1275 | 256793 | 16709 | 76 | 0 | 0.00 | 0 | 0 | 0 | 0.00 | 0 | 0 |
|  |  | sY1285 | 256800 | 16819 | 110 | 0 | 0.00 | 0 | 0 | 0 | 0.00 | 0 | 0 |
|  |  | G66117 | 229956 | 16857 | 38 | 0 | 0.00 | 0 | 0 | 0 | 0.00 | 0 | 0 |
|  |  | sY1276 | 256794 | 16976 | 119 | 0 | 0.00 | 0 | 0 | 0 | 0.00 | 0 | 0 |
|  | ξ | DYS198 | 24880 | 17009 | 33 | 0 | 0.00 | 0 | 0 | 0 | 0.00 | 0 | 0 |
|  |  | G65888 | 230633 | 17154 | 145 | 0 | 0.00 | 0 | 0 | 0 | 0.00 | 0 | 0 |
|  |  | DYS289 | 230633 | 17259 | 105 | 0 | 0.00 | 0 | 0 | 0 | 0.00 | 0 | 0 |
|  |  | G66096 | 229935 | 17417 | 158 | 0 | 0.00 | 0 | 0 | 0 | 0.00 | 0 | 0 |
|  |  | DYS200 | 25909 | 17442 | 25 | 0 | 0.00 | 0 | 0 | 0 | 0.00 | 0 | 0 |
|  |  | DYS199 | 60936 | 17534 | 92 | 0 | 0.00 | 0 | 0 | 0 | 0.00 | 0 | 0 |
|  |  | G66335 | 230322 | 17558 | 24 | 0 | 0.00 | 0 | 0 | 0 | 0.00 | 0 | 0 |
|  |  | G65961 | 230557 | 17657 | 99 | 0 | 0.00 | 0 | 0 | 0 | 0.00 | 0 | 0 |
|  |  | DYS84 | 149015 | 17795 | 138 | 0 | 0.00 | 0 | 0 | 0 | 0.00 | 0 | 0 |
|  | ξ | DYS201 | 4749 | 17806 | 11 | 0 | 0.00 | 0 | 0 | 0 | 0.00 | 0 | 0 |
|  |  | DYS202^φ^ | 2577 | 17870 | 64 | 0 | 0.00 | 0 | 0 | 0 | 0.00 | 0 | 0 |
|  |  | sY1227 | 243200 | 18501 | 631 | 0 | 0.00 | 0 | 0 | 0 | 0.00 | 0 | 0 |
|  |  | G66093 | 229932 | 18504 | 3 | 0 | 0.00 | 0 | 0 | 0 | 0.00 | 0 | 0 |
|  |  | sY1283 | 256799 | 19000 | 496 | 0 | 0.00 | 0 | 0 | 0 | 0.00 | 0 | 0 |
|  |  | sY1309 | 256813 | 19190 | 190 | 0 | 0.00 | 0 | 0 | 0 | 0.00 | 0 | 0 |
|  |  | sY1287 | 256802 | 19420 | 230 | 0 | 0.00 | 0 | 0 | 0 | 0.00 | 0 | 0 |
|  | ϕξ | DYS212 | 16762 | 19440 | 20 | 0 | 0.00 | 0 | 0 | 0 | 0.00 | 0 | 0 |
|  |  | G65966 | 230562 | 19553 | 113 | 0 | 0.00 | 0 | 0 | 0 | 0.00 | 0 | 0 |
|  |  | G09411 | 75359 | 19705 | 152 | 0 | 0.00 | 0 | 0 | 0 | 0.00 | 0 | 0 |
|  |  | DYS213 | 11538 | 19732 | 27 | 0 | 0.00 | 0 | 0 | 0 | 0.00 | 0 | 0 |
|  |  | G65880 | 230626 | 19751 | 19 | 0 | 0.00 | 0 | 0 | 0 | 0.00 | 0 | 0 |
|  |  | G66109 | 229948 | 19801 | 50 | 0 | 0.00 | 0 | 0 | 0 | 0.00 | 0 | 0 |
|  |  | G65879 | 230625 | 19952 | 151 | 0 | 0.00 | 0 | 0 | 0 | 0.00 | 0 | 0 |
|  |  | G42832 | 94741 | 19998 | 46 | 0 | 0.00 | 0 | 0 | 0 | 0.00 | 0 | 0 |
|  |  | G66375 | 230362 | 20054 | 56 | 0 | 0.00 | 0 | 0 | 0 | 0.00 | 0 | 0 |
| AZFb |  | G42829 | 94738 | 20110 | 56 | 0 | 0.00 | 0 | 0 | 0 | 0.00 | 0 | 0 |
|  |  | G65919^φ^ | 230664 | 20150 | 40 | 0 | 0.00 | 0 | 0 | 0 | 0.00 | 0 | 0 |
|  |  | G66542 | 230379 | 20232 | 82 | 0 | 0.00 | 0 | 0 | 0 | 0.00 | 0 | 0 |
|  |  | G65311 | 166394 | 20267 | 35 | 0 | 0.00 | 0 | 0 | 0 | 0.00 | 0 | 0 |
|  |  | G65292 | 166402 | 20312 | 45 | 0 | 0.00 | 0 | 0 | 0 | 0.00 | 0 | 0 |
|  |  | G65915 | 230660 | 20352 | 40 | 0 | 0.00 | 0 | 0 | 0 | 0.00 | 0 | 0 |
|  |  | SHGC-5449 | 49628 | 20392 | 40 | 0 | 0.00 | 0 | 0 | 0 | 0.00 | 0 | 0 |
|  | ϕξ | DYS215 | 26539 | 20525 | 133 | 0 | 0.00 | 0 | 0 | 0 | 0.00 | 0 | 0 |
|  |  | G65916 | 230661 | 20537 | 12 | 0 | 0.00 | 0 | 0 | 0 | 0.00 | 0 | 0 |
|  |  | sY1252 | 256788 | 20604 | 67 | 0 | 0.00 | 0 | 0 | 0 | 0.00 | 0 | 0 |
|  |  | G66152^φ^ | 229990 | 20800 | 196 | 0 | 0.00 | 1 | 0.01 | 0 | 0.00 | 1 | 0.004 |
|  |  | sY1253 | 256789 | 20851 | 51 | 0 | 0.00 | 0 | 0 | 0 | 0.00 | 0 | 0 |
|  |  | G66556 | 230394 | 20907 | 56 | 0 | 0.00 | 0 | 0 | 0 | 0.00 | 0 | 0 |
|  | ϕξ | DYS218 | 53243 | 20909 | 2 | 0 | 0.00 | 0 | 0 | 0 | 0.00 | 0 | 0 |
|  |  | RH69456 | 86083 | 20936 | 27 | 0 | 0.00 | 0 | 0 | 0 | 0.00 | 0 | 0 |
|  |  | M273 | 260467 | 21078 | 142 | 0 | 0.00 | 0 | 0 | 0 | 0.00 | 0 | 0 |
|  | ϕξ | DYS219 | 52297 | 21166 | 88 | 0 | 0.00 | 0 | 0 | 0 | 0.00 | 0 | 0 |
|  |  | G65893 | 230638 | 21329 | 163 | 0 | 0.00 | 0 | 0 | 0 | 0.00 | 0 | 0 |
|  |  | G65894 | 230639 | 21366 | 37 | 0 | 0.00 | 0 | 0 | 0 | 0.00 | 0 | 0 |
|  |  | G65935 | 229924 | 21413 | 47 | 0 | 0.00 | 0 | 0 | 0 | 0.00 | 0 | 0 |
|  |  | G65977 | 230573 | 21486 | 73 | 0 | 0.00 | 0 | 0 | 0 | 0.00 | 0 | 0 |
|  | ϕξ | DYS221 | 12002 | 21582 | 96 | 0 | 0.00 | 0 | 0 | 0 | 0.00 | 0 | 0 |
|  |  | G65978 | 230574 | 21650 | 68 | 0 | 0.00 | 0 | 0 | 0 | 0.00 | 0 | 0 |
|  |  | SHGC-9460 | 30391 | 21767 | 117 | 0 | 0.00 | 0 | 0 | 0 | 0.00 | 0 | 0 |
|  |  | G68330 | 230000 | 21805 | 38 | 0 | 0.00 | 0 | 0 | 0 | 0.00 | 0 | 0 |
|  | Ψξ | DYF74S1 | 44109 | 21836 | 31 | 0 | 0.00 | 0 | 0 | 0 | 0.00 | 0 | 0 |
|  | ϕξ | DYS223 | 21574 | 21837 | 1 | 0 | 0.00 | 0 | 0 | 0 | 0.00 | 0 | 0 |
|  | ξ | DYF81S1 | 24316 | 21885 | 48 | 0 | 0.00 | 0 | 0 | 0 | 0.00 | 0 | 0 |
|  | *ϕξ | DYS224 | 57476 | 21894 | 9 | 0 | 0.00 | 0 | 0 | 0 | 0.00 | 0 | 0 |
|  |  | SHGC-100328 | 170463 | 21919 | 25 | 0 | 0.00 | 0 | 0 | 0 | 0.00 | 0 | 0 |
|  |  | sY1302 | 256806 | 22068 | 149 | 0 | 0.00 | 0 | 0 | 0 | 0.00 | 0 | 0 |
|  |  | G65905 | 230650 | 22128 | 60 | 0 | 0.00 | 0 | 0 | 0 | 0.00 | 0 | 0 |
|  |  | sY1213 | 256771 | 22229 | 101 | 0 | 0.00 | 0 | 0 | 0 | 0.00 | 0 | 0 |
|  | ξ | DYS230 | 14237 | 22316 | 87 | 0 | 0.00 | 0 | 0 | 0 | 0.00 | 0 | 0 |
|  |  | sY1294 | 256805 | 22345 | 29 | 0 | 0.00 | 0 | 0 | 0 | 0.00 | 0 | 0 |
|  |  | G66018 | 230614 | 22699 | 354 | 0 | 0.00 | 0 | 0 | 0 | 0.00 | 0 | 0 |
|  |  | G66105 | 229944 | 22752 | 53 | 0 | 0.00 | 0 | 0 | 0 | 0.00 | 0 | 0 |
|  |  | SHGC-102574 | 168906 | 22801 | 49 | 0 | 0.00 | 0 | 0 | 0 | 0.00 | 0 | 0 |
|  |  | sY1197 | 243191 | 22862 | 61 | 0 | 0.00 | 0 | 0 | 0 | 0.00 | 0 | 0 |
| AZFc |  | sY1192 | 243189 | 23211 | 349 | 0 | 0.00 | 0 | 0 | 0 | 0.00 | 0 | 0 |
|  |  | sY1291 | 243206 | 23843 | 632 | 4 | 0.02 | 4 | 0.042 | 0 | 0.00 | 8 | 0.03 |
|  |  | Y-DAZ3 | 242966 | 25247 | 1404 | 12 | 0.07 | 9 | 0.094 | 0 | 0.00 | 21 | 0.077 |
|  |  | sY1201 | 243193 | 26795 | 1548 | 0 | 0.00 | 0 | 0 | 0 | 0.00 | 0 | 0 |
|  |  | DYF91S1^φ^ | 42302 | 26933 | 138 | 0 | 0.00 | 0 | 0 | 0 | 0.00 | 0 | 0 |
|  |  | G66111 | 229950 | 27093 | 160 | 0 | 0.00 | 0 | 0 | 0 | 0.00 | 0 | 0 |
|  |  | sY1246 | 256783 | 27145 | 52 | 0 | 0.00 | 0 | 0 | 0 | 0.00 | 0 | 0 |

φ Markers that were redesigned to validate initial negative results

* Markers also used in the study by (Frydelund-Larsen et al., 2003)

ϕ Markers also used in the study by (Lutke Holzik et al., 2005)

Ψ Markers also used in the study by (Bor et al., 2006)

ξ Markers used in studies by (Bianchi et al., 2002;Richard et al., 2004;Bianchi et al., 2006)
